# Daily Activity Rhythms of Animals in the Southwest Mountains, China: Influences of Interspecific Relationships and Seasons

**DOI:** 10.3390/ani14192842

**Published:** 2024-10-02

**Authors:** Qiuxian Li, Qian Zhang, Qingsong Jiang, Huaqiang Zhou, Zejun Zhang, Hong Zhou, Wei Wei, Mingsheng Hong

**Affiliations:** 1 Liziping Giant Panda’s Ecology and Conservation Observation and Research Station of Sichuan Province (Science and Technology Department of Sichuan Province), China West Normal University, Nanchong 637009, China; 13527330212@163.com (Q.L.); 18919552856@163.com (Q.J.); 19161067250@163.com (H.Z.); zhangzj@ioz.ac.cn (Z.Z.); zhouhong1026@163.com (H.Z.); weidamon@163.com (W.W.); 2 Key Laboratory of Southwest China Wildlife Resources Conservation (Ministry of Education), China West Normal University, Nanchong 637009, China; 3 Appraisal Center for Environment and Engineering, Ministry of Ecology and Environment, Beijing 100006, China; zhangqian@acee.org.cn

**Keywords:** camera traps, coexistence, daily activity rhythm, season, interspecific relationship

## Abstract

**Simple Summary:**

Predation relationships and competition relationships shape interspecies coexistence in wild animal communities. Studying the temporal and spatial activity patterns of wild animals is crucial for understanding their behavior, species interactions, and resource requirements. We evaluated the spatiotemporal overlap between 15 different dominant species in the southwestern mountains of China, including Carnivora (such as *Panthera pardus* and *Lynx lynx*), Artiodactyla (such as *Moschus* spp. and *Rusa unicolor*), Primate (*Macaca mulatta*), and Galliformes (*Crossoptilon crossoptilon*, *Ithaginis cruentus*). We found that different species exhibit different activity patterns to reduce intense resource competition, with competition being more intense in cold seasons than warm seasons. This may be due to abundant resources in summer but scarce food in winter, as well as increased energy demands during cold seasons, as expected using physiology theory.

**Abstract:**

Temporal and spatial factors regulate the interactions between apex predators, mesocarnivores, and herbivores. Prey adjust their activity patterns and spatial utilization based on predator activities; in turn, predators also adapt to the activities of their prey. To elucidate the factors influencing the daily activity rhythms of animals, 115 camera traps were established from September 2019 to June 2023 to assess the influences of interspecific relationships and seasons on the daily activity rhythms of animals in the southwest mountains of China. The species captured by the cameras included six Carnivora (such as *Panthera pardus* and *Lynx lynx*), six Artiodactyla (such as *Moschus* spp. and *Rusa unicolor*), one Primate (*Macaca mulatta*), and two Galliformes (*Crossoptilon crossoptilon*, *Ithaginis cruentus*). The results demonstrated that the 15 species exhibited different activity rhythms and peak activities to reduce intense resource competition. There were differences in the species’ activity rhythms in different seasons, with competition among different species being more intense in the cold season than in the warm season. In predation relationships, the overlap coefficient in the cold season exceeded that of the warm season, possibly due to the abundant resources in summer and food scarcity in winter. In competitive relationships, 15 pairs of species exhibited significantly higher overlap coefficients in the cold season compared to the warm season, possibly due to increased demands for energy during the cold period or seasonal changes in predatory behavior. By analyzing the daily and seasonal activity patterns of dominant species in the study area, temporal niche overlaps were established to compare the competition levels between species. These findings indicate that the activity rhythms of the animals in this area not only result from evolutionary adaptation but are also influenced by season, food resources, and interspecific relationships (predation and competition). Thus, efforts should be made to reduce human interference, protect food resources in the winter, and monitor animals’ interspecific relationships to protect animal diversity and maintain the stability of the ecosystem in this biodiversity hotspot in China.

## 1. Introduction

Surveying biodiversity among communities is a crucial strategy for understanding local wildlife populations and distributions, which can inform the development of effective conservation management measures [1,2]. Mammals are a vital component of biodiversity, playing significant roles in the food chains of ecosystems at various spatial scales [3]. The activity rhythm of a species, defined as the intensity and pattern of activity within a fixed period, relates to the animal’s nutritional status and survival pressures. These activity rhythms are a key focus in behavioral ecological research [4,5].

Variations in the temporal and spatial niches of carnivores is crucial for reducing competition intensity [6]. When carnivores have similar spatial characteristics and functional traits, differences in their activity rhythms can effectively promote their coexistence within a region [7]. For instance, the Mexican gray fox (*Urocyon cinereoargenteus*) and the white-nosed coati (*Nasua narica*), which have highly similar diets and habitat preferences, exhibit significant differences in their activity patterns to reduce competition [8]. The sun bear (*Helarctos malayanus*), yellow-throated martens (*Martes flavigula*), and other small carnivores in Indonesia reduce the negative effects of competition by flexibly altering their temporal activity patterns [9].

The study of herbivorous animal behavior includes examining their home ranges, activity patterns, foraging behaviors, seasonal migrations, and so on [10,11,12]. Herbivores exhibit high species richness and abundance, with diverse resource and habitat uses. The activity patterns of herbivores show significant variation across different months and times of the day. For example, wild boars (*Sus scrofa*) in the Qinling Mountains are more active from August to October, while dwarf musk deer (*Moschus berezovskii*) are more active in December [13]. However, previous research on herbivore behavior has primarily focused on individual species, with less attention given to the interactions and activity patterns among different species and across different seasons [13,14].

To better protect endangered species, it is crucial to understand the coexistence mechanisms of sympatric wildlife [15]. Ecological differences [16,17], foraging strategies [18], and activity patterns [19] are important factors related to species coexistence. There are variations in the temporal niches utilized by different species [20], and studies on the differentiation of these niches are increasing [21,22]. An understanding of these variations in temporal niches contributes to our understanding of interspecies relationships [23,24]. Habitat, interspecies relationships, and food are key factors affecting daily activity patterns [25,26]. Seasonal changes in environmental conditions significantly impact animal activity patterns [22,27]. For instance, differences in light intensity and precipitation between the cold and warm seasons affect vegetation growth and may alter the activity patterns of wild herbivores and, consequently, carnivores [19]. Such seasonal differences also cause distinct seasonal differences in the spatial distribution of elephants and deer [14].

The presence of predators initiates anti-predatory behaviors in prey, including an increased frequency or duration of vigilance postures, enlarged group sizes, and the adoption of behaviors that minimize temporal or spatial overlap with predators [28,29]. Prey adjust their feeding times based on the likelihood of predator encounters [30,31]. Conversely, predators adapt their hunting behaviors and activity patterns to align with the activity patterns of their prey [31,32]. Thus, the spatial and temporal overlap between predators and prey depends on a balance between the need to acquire food (which is important for both) and the need to avoid predation (critical only for prey) [33].

Infrared camera technology is currently the most effective method for monitoring wildlife. It is capable of operating uninterrupted 24 h a day and can collect information on animal populations, numbers, and behaviors. It has a long history, both domestically and internationally, with the earliest reports of the technology dating back to 1927. Infrared camera technology gradually matured in the 1990s and is now widely used in wildlife surveys [34]. Here, as part of a long-term camera-trapping study on rare species in the southwest mountains, China, infrared cameras were used to elucidate the factors influencing the daily activity rhythms of animals. The objectives of this research were to (1) reveal the daily activity rhythm types of dominant species in the southwest mountains, China, (2) compare the differences in the daily activity rhythms of dominant species among the different seasons, and (3) assess the influences of interspecific relationships and seasons on the daily activity rhythms of animals. It was hoped that the findings would provide a reference for the conservation and management of animal diversity in this biodiversity hotspot in China.

## 2. Materials and Methods

### 2.1. Study Area

**Figure 1:**
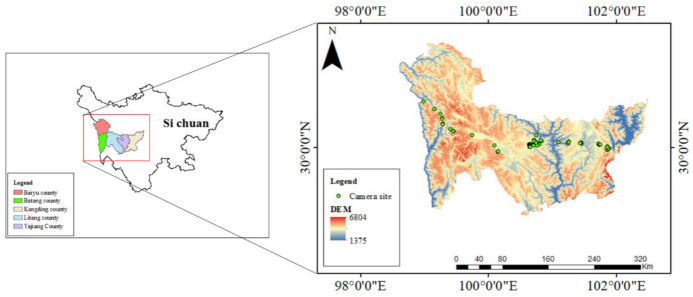
Camera locations (green triangles) in southwest mountains, Sichuan, China.

This research was conducted in the southwest mountains, Sichuan, China, which are located between 98.41° E to 102.36° E longitude and 22.51° N to 28.14° N latitude (Figure 1). This region is within one of the world’s 36 biodiversity hotspots, boasting one of the richest subtropical forest ecosystems globally [35]. Major rivers such as the Jinsha, Yalong, and Dadu Rivers flow roughly parallel from north to south through this region, crossing major mountains like the Gongga Mountains, Daxue Mountains, and Shaluli Mountains. The area is home to several threatened mammal species facing significant disturbance, including the leopard (*Panthera pardus*) and Eurasian lynx (*Lynx lynx*), among others [35,36]. The altitude of infrared camera locations ranged from 3127 m to 4337 m, encompassing vegetation types such as mixed coniferous broad-leaved forests, cold-temperate conifer forests, high-altitude shrublands and meadows, and so on. The research area has a plateau mountain climate, with an annual average temperature ranging from 0.6 °C to 16.3 °C and an annual total precipitation ranging from 417.8 mm to 935.8 mm. The rainfall is concentrated in the summer months, from June to August. From November to April of the following year, the region was mostly covered by snow. Based on the climatic characteristics of the research area, the year was divided into a warm season and a cold season (warm season: 1 May–30 September; cold season: 1 October–30 April).

### 2.2. Experimental Design

From September 2019 to June 2023, a non-invasive camera-trapping survey was conducted in the study area. We installed 115 infrared cameras (model LTI-6511, Shenzhen, China) in areas with higher animal activity (such as ridges, animal paths, and near water sources) based on survey results, transect surveys, and accessibility principles, with each camera spaced at least 500 m apart; baits were not used. The cameras were positioned approximately 40–80 cm above ground level, with the aim of minimizing direct sunlight exposure, ensuring a clear field of view, and avoiding areas visibly disturbed by human activities. Additionally, we adjusted camera locations based on the camera trap results during data collection to ensure the thorough monitoring of rare wildlife in different areas. The camera settings were as follows: sensitivity level: “medium”; capture mode: “photo + video”; three consecutive photos and a 10 s video recorded upon trigger; and 10 s interval between triggers. The longitude, latitude, and habitat information of each camera were recorded. The batteries and memory cards of each camera were replaced every 5–6 months, and any damaged cameras were promptly replaced. Due to the difficulty in distinguishing between *Moschus chrysogaster* and *Moschus berezovskii* from the camera images, all photos of *M. chrysogaster* and *M. berezovskii* were classified under the family *Moschus* spp. [37].

### 2.3. Statistical Analysis

Photos of the same species taken at the same camera site with an interval of 30 min were considered as one independent detection [38]. The frequency of these independent detections can serve as an indicator of how likely a specific species is to visit a site [39]. The relative abundance index (RAI), which is the average number of independent detections per camera day, was used to assess the spatial overlap between target species at each site [40]. The RAI was calculated using the following formula:

RAIi,j = Ni,j/Di

where RAIi,j indicates the relative abundance index of species i at trap j; Ni,j indicates the effective detection number of species i at trap j; and Di indicates the effective working days of the camera at trap j [38].

Sunrise Sunset Times Lookup (https://sunrise.maplogs.com/) was used to determine the precise times of sunrise and sunset in the study area [41]. Dawn (06:00–08:00) and dusk (18:00–20:00) were defined as one hour before and after sunrise and sunset, respectively. The intervening time periods were categorized as daytime (08:00–18:00) and nighttime (20:00–06:00) [42]. All observations were classified into daytime (<15% nighttime observations), nighttime (>85% nighttime observations), mostly daytime (15–35% nighttime observations), mostly nighttime (65–85% nighttime observations), dusk (50% observations during dusk), and cathemeral (active during both day and night) [43,44].

To estimate the activity patterns of the animals, the “overlap” package in R 4.3.2 was utilized [45]. Each species’ detection times were converted from clock time to relative solar time. The daily activity patterns of each target species were analyzed using kernel density estimation based on the timing information from each independent record [45]. The temporal niche overlap index, ranging from 0 (no overlap) to 1 (complete overlap), was used to assess the temporal overlap of the activity rhythms of different species in the warm and cold seasons. For sample sizes greater than 75, the D4 estimator was used; for sample sizes less than 75, the D1 estimator was applied. An overlap coefficient greater than 0.75 indicated high overlap, a coefficient between 0.5 and 0.75 indicated moderate overlap, and a coefficient less than 0.5 indicated low overlap [46].

To assess the effects of the seasons on predation and competition [42], the bootstrap method with 10,000 iterations was used to calculate the 95% confidence interval [45]. Given the circular distribution of the activity data, the “circular” package in R [47] was utilized. Additionally, the nonparametric Watson–Wheeler test of homogeneity of means was employed to assess whether there were significant differences in the species distributions across the different seasons [48].

## 3. Results

### 3.1. Species Overview

Over 37,112 camera days, a total of 11,440 independent photographs were obtained from the 115 camera locations. The herbivores (including two Galliformes) that appeared more than 50 times independently and the carnivores that appeared more than 20 times independently were documented and analyzed (Table 1). Among them, herbivores were captured in 5964 independent detections (3931 in the warm season and 2033 in the cold season), carnivores were captured in 414 independent detections (255 in the warm season and 159 in the cold season), Galliformes were captured in 925 independent detections (526 in the warm season and 399 in the cold season), and omnivores were captured in 1043 detections (580 in the warm season and 463 in the cold season).

### 3.2. Daily Activity Rhythm Types

**Figure 2:**
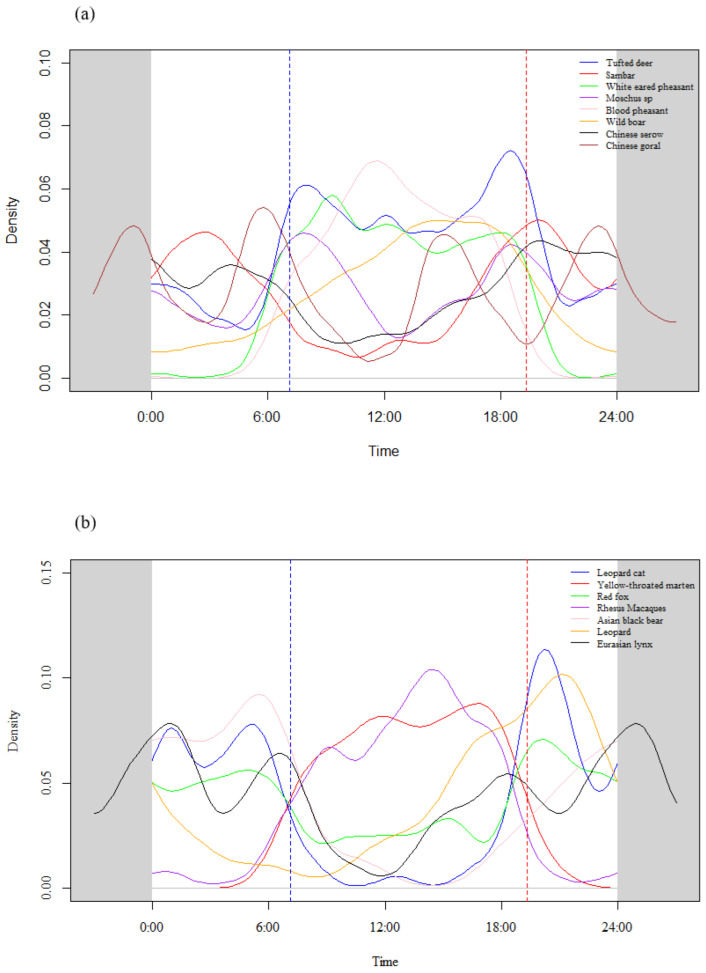
Kernel densities of daily activity of fifteen animals in southwest mountains, China. (**a**) Six ungulate species and two Galliform species; (**b**) six Carnivora species and one Primate species. Two vertical lines represent the average time of sunrise and sunset.

Mammals were active during both the day and night (Figure 2 and Table 2). Tufted deer, *Moschus* spp., and wild boars were primarily diurnal, with tufted deer and *Moschus* spp. showing multiple activity peaks and a wide distribution of activity times, while wild boars had only one peak at around 3:00 PM (Figure 2a and Table 2). *Rhesus macaque* and yellow-throated martens exhibited bimodal activity patterns, with peaks concentrated during the daytime; they were categorized as diurnal species (Figure 2b and Table 2). Leopard cats and Asiatic black bears had three activity peaks, with peaks occurring before 6:00 AM and after 6:00 PM; thus, they were classified as predominantly nocturnal species (Figure 2b and Table 2). The remaining species, such as leopards, Eurasian lynx, and red foxes, exhibited activity during both the day and night. White-eared pheasants and blood pheasants were diurnal animals (Figure 2a and Table 2). Their activity displayed a bimodal pattern, with activity peaks appearing higher during the dawn than dusk, although white-eared pheasants had a broader activity distribution than blood pheasants (Table 2). When comparing the activity timing ratios of all species, Asiatic black bears, the Eurasian lynx, and white-eared pheasants were most active at dawn; blood pheasants, yellow-throated martens, and macaque had the highest activity ratios during the day and dusk; and leopard cats, Asiatic black bears, and sambar deer were most active at night (Figure 2 and Table 2).

### 3.3. Seasonal Variation in Daily Activity Patterns

**Figure 3:**
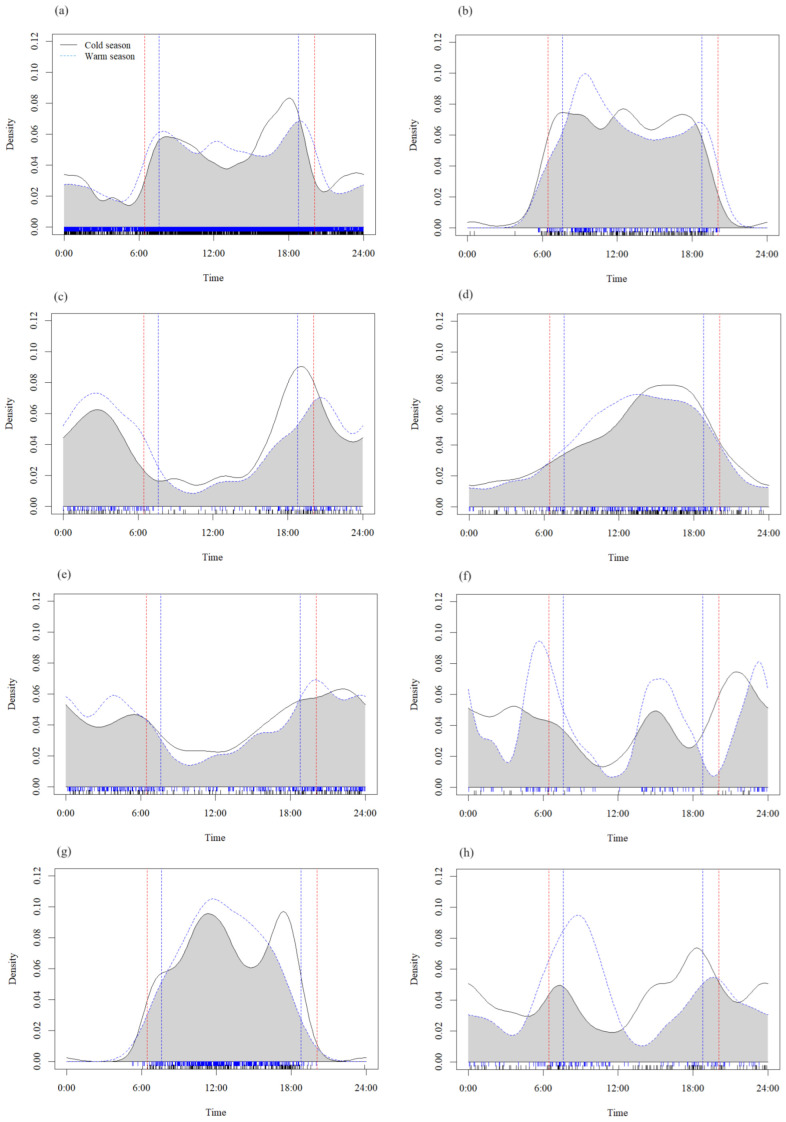
Daily activity rhythms of six ungulate species and two Galliform species between warm and cold seasons in the southwest mountains, China. (**a**) Tufted deer, (**b**) white-eared pheasant, (**c**) sambar, (**d**) wild boar, (**e**) Chinese serow, (**f**) Chinese goral, (**g**) blood pheasant, and (**h**) *Moschus* spp. The blue dotted line: the warm season; the black solid line: the cold season; the red line: the time of sunrise (**left**) and sunset (**right**) in the warm season; and the blue line: the time of sunrise (**left**) and sunset (**right**) in the cold season.

**Figure 4:**
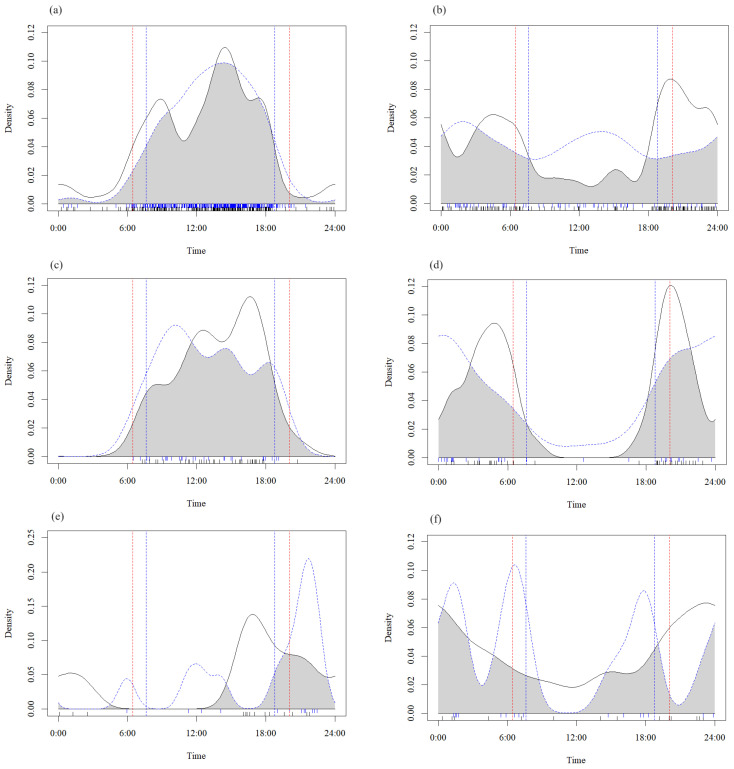
Daily activity rhythms of six Carnivora species and one Primate species between warm and cold seasons in the southwest mountains, China. (**a**) Macaque, (**b**) red fox, (**c**) yellow-throated marten, (**d**) leopard cat, (**e**) leopard, (**f**) Eurasian lynx. The black bear is not included as it is largely inactive during the cold season. Blue dotted line: warm season; black solid line: cold season; the red line: the time of sunrise (**left**) and sunset (**right**) in the warm season; and the blue line: the time of sunrise (**left**) and sunset (**right**) in the cold season.

The animal activity rhythms varied across the seasons (Figure 3 and Figure 4). The Watson–Wheeler test revealed significant differences in the daily activity patterns of tufted deer (*p* < 0.01), blood pheasants (*p* < 0.05), and *Moschus* spp. (*p* < 0.05) between the warm and cold seasons. In the warm season, the first peak of tufted deer activity occurred earlier, and the second peak shifted later compared to the cold season (Figure 3a). Blood pheasants exhibited only one activity peak in the warm season but two in the cold season (Figure 3g). *Moschus* spp. displayed two activity peaks in both seasons; in the warm season, the first peak was significantly more intense than the second, and the opposite was true in the cold season (Figure 3h).

Other species showed no significant differences in activity rhythms, but there were changes in their patterns. For example, macaque had only one activity peak in the warm season but two in the cold season, with the peaks occurring earlier in the cold season than in the warm season (Figure 4a).

### 3.4. Seasonal Variation in Interspecific Relationships

In predator–prey pairs (Table 3 and Table S1), there were three highly overlapping pairs and three moderately overlapping pairs (Table S2). The highly overlapping pairs were yellow-throated martens and white-eared pheasants (Δ = 0.88, *p* = 0.16), yellow-throated martens and blood pheasants (Δ = 0.91, *p* = 0.35), and red foxes and *Moschus* spp. (Δ = 0.78, *p* < 0.01), with only red foxes and *Moschus* spp. showing significant differences. The three moderately overlapping pairs were yellow-throated martens and *Moschus* spp. (Δ = 0.61, *p* < 0.01), yellow-throated martens and tufted deer (Δ = 0.74, *p* < 0.01), and red foxes and tufted deer (Δ = 0.68, *p* < 0.01). All three moderately overlapping pairs showed significant differences within each pair. Wild boars and Asian black bears are omnivorous animals with a broad diet that mainly consists of nuts, berries, and tender leaves. In some cases, they also prey on small animals such as pheasants [42]. There was a high degree of overlap between wild boars and two species of pheasants, the white-eared pheasant (Δ = 0.82, *p* < 0.01) and the blood pheasant (Δ = 0.78, *p* < 0.01), with significant differences observed. The overlap between the Asiatic black bear and pheasants was relatively low; leopards and ungulates showed moderate overlap.

Regarding the temporal overlap between competing species (Table 4 and Table S3), seven pairs showed high overlap (Table S2), while five pairs showed moderate overlap (Table S2). The seven species with high overlap were red foxes and leopard cats (Δ = 0.78, *p* = 0.03), tufted deer and *Moschus* spp. (Δ = 0.86, *p* < 0.01), sambar and *Moschus* spp. (Δ = 0.75, *p* < 0.01), sambar and Chinese goral (Δ = 0.86, *p* = 0.49), *Moschus* spp. and Chinese serow (Δ = 0.80, *p* < 0.01), Chinese serow and Chinese goral (Δ = 0.78, *p* = 0.04), and white-eared pheasants and blood pheasants (Δ = 0.85, *p* < 0.01). Aside from sambar and Chinese goral, all other competing pairs had significant differences. Among the five pairs with moderate overlap, four pairs had significant differences: tufted deer and sambar (Δ = 0.65, *p* < 0.01), tufted deer and Chinese goral (Δ = 0.67, *p* < 0.01), tufted deer and Chinese serow (Δ = 0.70, *p* < 0.01), and sambar and Chinese goral (Δ = 0.68, *p* < 0.01) (Table S2). The highest overlap coefficient was between wild boars and white-eared pheasants (Δ = 0.85, *p* < 0.01), followed by white-eared pheasants and tufted deer (Δ = 0.79, *p* < 0.01). Blood pheasants had a moderate overlap with tufted deer (Δ = 0.69, *p* < 0.01) (Table S1).

### 3.5. Influences of the Seasons on Interspecific Relationships

For the predator–prey relationship, the overlap coefficients of diurnal activity between yellow-throated martens and blood pheasants, yellow-throated martens and white-eared pheasants, and red foxes and *Moschus* spp. were all higher in the cold season than in the warm season (Table 3). In contrast, the overlap coefficients of the other species were higher in the warm season than in the cold season (Table 3). The overlap between yellow-throated martens and tufted deer, and between red foxes and tufted deer, was high in the warm season and moderate in the cold season, while it was the opposite for red foxes with *Moschus* spp. (Table 3). In addition, during the cold season only, there were significant differences in overlap times between red foxes and tufted deer, as well as between red foxes and *Moschus* spp. (Table 3). The overlap coefficients between wild boars and white-eared pheasants and between wild boars and blood pheasants were greater in the warm season than in the cold season. Leopards and ungulates showed moderate overlap, with the cold season having a higher overlap coefficient than the warm season (Table S3).

In competitive relationships (Table 4), the daily activity overlap coefficients between yellow-throated martens and red foxes, yellow-throated martens and leopard cats, and sambar and Chinese serow were higher in the warm season than in the cold season (Table 4). The overlap coefficients of the other species were higher in the cold season compared to the warm season (Table 4). Red foxes and leopard cats, sambar and *Moschus* spp., sambar and Chinese goral, *Moschus* spp. and Chinese serow, *Moschus* spp. and Chinese goral, and Chinese serow with Chinese goral exhibited high overlap during the cold season but moderate overlap during the warm season (Table 4). Additionally, significant differences were observed in the time overlaps during the warm season only between red foxes and leopard cats, sambar and *Moschus* spp., sambar and Chinese goral, *Moschus* spp. and Chinese serow, *Moschus* spp. and Chinese goral, Chinese serow and Chinese goral, and white-eared pheasants and blood pheasants (Table 4).

There were no significant differences in the overlap coefficients between Galliformes and Artiodactyla in the cold and warm seasons. The overlap coefficients between white-eared pheasants and tufted deer were highly overlapping in both the cold and warm seasons. There were no significant differences in the overlap coefficients between wild boars and Artiodactyla in both the cold and warm seasons. In the warm season, the overlap coefficient between wild boars and *Moschus* spp. was the highest, while in the cold season, it was highest with tufted deer. The overlap coefficients between wild boars and small carnivores were relatively higher in the warm season than in the cold season, with the highest coefficients observed with yellow-throated martens in both the cold and warm seasons (Table S3).

## 4. Discussion

Wild animal species have long been an important community for biodiversity monitoring and research. The population status and dynamics of many important species are still unclear; thus, monitoring and research need to be strengthened [49]. Through intensive camera-trap surveys, we comprehensively explored the spatial and temporal activity patterns of various wild mammal species in the southwest mountains, China, enabling us to quantify the behavior of wild mammals and collect data on multiple species with less time, effort, and disturbance to wildlife [50]. From September 2019 to July 2023, the animals with the highest independent detections were Acrodactyla, such as the tufted deer, wild boar, and Chinese serow, followed by Primates and Galliformes; the lowest independent detections were among Carnivora (Table 1). A structure comprising more primary consumers and fewer secondary consumers is considered to be conducive to the stability of food chains and food webs and is also conducive to the balance of the ecosystem. Generally, more detections of Artiodactyla and Primates occurred during the warm season, but more Carnivora were found during the cold season (Table 1). These results may reflect the abundant food resources in the warm season, which facilitate the foraging and breeding activities of herbivores; these animals can adjust their foraging behavior, animal physiology, movement ecology, and survival strategies according to environmental changes, such as temperature, precipitation, and food supply, in different seasons [13,51]. However, large- and medium-sized carnivores were more abundant in winter than in the warm season. This may be because there was less disturbance in cold conditions at high altitudes.

Activity rhythms are part of the evolutionary adaptation of animals, serving as a response to environmental changes. The daily activity rhythm is considered a determining factor in an animal’s activity rhythm, with food, social structure, gender, and competitive predation relationships playing dominant roles. Over time, natural selection shapes the activity rhythm of each species [52], and interspecific competition also plays a role [53]. Research shows that herbivores choose different times to be active to avoid competition, while carnivores feed at different times to promote interspecies coexistence [9]. The current results indicate that white-eared pheasants and blood pheasants are active during the day, with two peaks in activity. Our findings are similar to those of Jia et al. [42]. The physical and nutritional needs of these pheasants may be the main factors influencing their activity patterns. This requires verification in further research [54].

Distinct differences were apparent in the activity patterns of various mammals (Figure 2 and Table 2). The macaque and yellow-throated marten were primarily active during the day. *Moschus* spp., tufted deer, and wild boar were mostly diurnal, with *Moschus* spp. exhibiting activity peaks at 8:00 AM. This is in contrast to the findings of Li et al. [13], who found that their activity occurred between 4:00 PM and midnight, with a peak at 6:00 PM in the Qinling Mountains. Tufted deer exhibited an increase in activity at 6:00 AM and a decrease by 6:00 PM, similar to the findings of Tian et al. [19] in the Wanglang National Nature Reserve. The peak in wild boar activity was between 2:00 PM and 4:00 PM. This is different from Rossa et al. [33], who found two activity peaks with a later timing in the Mediterranean region.

Black bears and leopards were more active at night, with activity peaks before 6:00 AM and after 8:00 PM, indicating that they were nocturnal. The other mammals, i.e., red fox, sambar, Chinese goral, Chinese serow, leopard, and Eurasian lynx, exhibited both diurnal and nocturnal activities (Figure 2 and Table 2). The activity patterns of small carnivores were similar to those found by Nakabayashi et al. [9] in Borneo, with leopard cats and yellow-throated martens exhibiting similar rhythms. Our findings in relation to black bears differ significantly from those of Jia et al. [13] in the Taipinggou National Nature Reserve, where black bears were active both day and night, possibly due to the low number of bear photos captured. The activity rhythms of ungulates were generally similar, but some variation in their daily activity patterns exist due to geographical differences [55,56].

Research on seasonal daily activity patterns contributes to our understanding of the temporal ecological niches of animals [57]. Mammalian activity patterns exhibit significant overlap and distinct differences across different seasons [56]. In addition, the activity patterns change with the seasons due to resource availability and climatic conditions, which may reflect the intensity of competition among coexisting species [50,58]. The current study revealed seasonal differences in the activity rhythms of tufted deer, blood pheasants, and *Moschus* spp. (Figure 3). Tufted deer exhibited significant seasonal differences in activity levels, coinciding with sunrise and sunset [56]. Blood pheasant activity peaks also shifted with temperature changes; a single peak occurred in the warm season, aligning with the morning peak of the cold season at around 11:00 AM (Figure 3). However, activity significantly increased between 5:00 PM and 6:00 PM in the cold season, forming a second peak, possibly due to the extended cold period requiring increased energy intake. *Moschus* spp., which are crepuscular, exhibited noticeable differences in peak activities between the cold and warm seasons. With rising temperatures, the activity of most animals increased in the morning and decreased at night, indicating that seasonal variation affects the foraging and survival strategies of most animals [59].

The interaction between predators and prey involves complex interspecific behaviors, with prey showing avoidance strategies towards predators. According to optimal foraging theory, both predators and prey seek strategies that are most advantageous for survival and reproduction, balancing energy costs and opportunities for foraging [60]. In predation relationships, there were higher degrees of overlap between the yellow-throated marten and both the white-eared pheasant and blood pheasant; their activity rhythms did not show significant differences (Table 3 and Table S2). This may be because the white-eared pheasant and both the blood pheasant and yellow-throated marten are all diurnal species (Table 2), and predators will also adjust their activity rhythms according to the peak activity of their prey [33]. The red fox overlapped highly with *Moschus* spp. and moderately with the tufted deer (Table 3 and Table S2). However, there were significant differences in the activity rhythms of the red fox and these other species. This may be because the red fox has a broader diet, also preying on some birds, invertebrates, rodents, and so on. Moreover, we obtained photos of wild boars coexisting with the two pheasants, with higher coefficients of daily activity overlap (Table S3). This may be because omnivorous animals have broad diets (including tubers, fruits, earthworms, pheasants, etc.) [61]. Lastly, there were lower coefficients of daily activity overlap among other large-body carnivores (such as the Eurasian lynx, black bear, and leopard) and their prey. This may be due to their limited populations and independent detections (Table 1). Therefore, further continuous long-term investigations and monitoring of these key apex predators should be performed in the future.

In competitive relationships, there was a high degree of overlap between red foxes and leopard cats, but with significant differences in their daily activity rhythms (Table 4). This indicates that red foxes and leopard cats alleviate competition by avoiding direct encounters [9], and carnivores also reduce competition by changing their dietary choices [33]. For instance, when wolves and red foxes coexist in one area, the diet of red foxes comprises mainly invertebrates and fruits; however, when wolves are absent from the area, the number of ungulates in the diets of red foxes increases threefold despite no significant change in the activity rhythm of ungulates [62].

For ungulates, temporal avoidance can reduce competition and promote species coexistence [63]. The majority of ungulate species (excluding wild boars) were active during the day and at dusk (Table 4), showing a clear bimodal pattern [64,65]. Generally, the peak activity of ungulates occurs at dusk when temperatures are relatively lower, and humidity is also lower. This is conducive to foraging and slow movement, thereby reducing overall activity and energy expenditure, especially in midsummer [65]. This indicates that solar radiation may be the primary environmental factor influencing the daily activity patterns of wild ungulates [66]. The coefficients of daily activity overlap between wild boars and other ungulate animals were relatively lower (Table S1). This may be because wild boars are omnivorous animals with a broader diet, including plants and animals [61]. Therefore, wild boars have stronger adaptability compared to other ungulate animals [13]. The two species of pheasants had high degrees of overlap in their habitats (Table 4), as well as similar feeding habits and habitat preferences. For example, the two pheasants usually engage in group activities to forage and avoid predators [42]. However, the mechanism of their coexistence requires further study.

Some medium-body carnivores (yellow-throated marten, red fox) prey on small ungulates and pheasants, exhibiting higher overlap coefficients in the cold season compared to the warm season (Table 3). Further, these carnivores also hunt small rodents, small birds, and reptiles. During the warm season, animal activity levels increase, and their diet expands, leading to a relatively reduced overlap coefficient [9]. There were also competitive relationships among the small- and medium-body carnivores, with the highest overlap coefficient between the red fox and leopard cat (Table 3; Δ = 0.78, *p* = 0.03). The overlap coefficient of competition in the cold season was also significantly higher than that in the warm season (Table 4). This may be because the ranging diets of red foxes and leopard cats in the study area are similar, and their prey activity levels decrease during the cold season, leading to intense competition between them [67]. Ungulates showed higher activity levels in the warm season than in the cold season, similar to previous research [67,68,69]. The low temperatures in the cold season reduce activity, which saves energy, and animals spend more time digesting less food [13]. However, the overlap coefficient was higher in the cold season, possibly due to food scarcity in winter within high-altitude areas in the southwest mountains, China, leading to more intense competition among ungulates. In the cold season at high altitudes, temperatures are low, and food is scarce. Some herbivores (ungulates, pheasants) may migrate to lower altitudes with abundant food resources. Furthermore, the two species of pheasants had a high degree of overlap throughout the year, especially in the cold season. This is related to their similar diet, habitat selection, and group activities [42].

## 5. Conclusions

This comprehensive study analyzed the factors influencing the daily activity rhythms of animals in the southwest mountains, China, using infrared camera technology. The southwest mountains, China, is a region rich in rare and endangered animal resources. The predominant animals observed in this survey were ungulates. Ungulates can impact the structure and function of ecosystems and serve as important ecological indicators of land ecosystem health [68]. Activity rhythms are not only the result of animal evolution and adaptation but are also influenced by environmental changes such as temperature and climate (i.e., season). Furthermore, food, social structure, interspecific relationships (such as predation and competition), and human interference can also affect the activity rhythms of species. Understanding the activity patterns of wildlife is crucial for ecosystems [13,69]. Therefore, there is an ongoing need for the long-term standardized monitoring of wildlife and habitats in this area. Moreover, given that different animals with varied feeding habits exhibited different activity patterns in the warm and cold seasons, categorized management should be implemented. For example, ungulates in the research area, such as musk deer and tufted deer, are sensitive to the environment and have a weak anti-interference ability. Thus, human interference should be minimized during the warm season, as this season is characterized by abundant food and a high activity rate of these herbivores in this region. Moreover, there is a need to protect food resources in the winter when food is scarce. Further continuous long-term investigations and the monitoring of key apex predators and their interspecific relationships should be performed in the future. Together, the implementation of these measures can help protect animal diversity and the stability of the ecosystem in this biodiversity hotspot in China.

## Figures and Tables

**Table 1:** The number of independent detections of fifteen animals in the southwest mountains, China, in two different seasons.

Orders	Species	Diets	Total	Cold Season	Warm Season
Carnivora	*Vulpes vulpes*(red fox)	Carnivore	215	146	69
Carnivora	*Prionailurus bengalensis* (leopard cat)	Carnivore	73	44	29
Carnivora	*Martes flavigula* (yellow-throated marten)	Carnivore	67	36	31
Carnivora	*Lynx lynx* (Eurasian lynx)	Carnivore	33	15	18
Carnivora	*Ursus thibetanus* (Asiatic black bear)	Omnivore	28	23	5
Carnivora	*Panthera pardus* (leopard)	Carnivore	26	14	12
Artiodactyla	*Elaphodus cephalophus* (tufted deer)	Herbivore	4874	1615	3259
Artiodactyla	*Sus scrofa* (wild boar)	Omnivore	467	247	220
Artiodactyla	*Capricornis milneedwardsii* (Chinese serow)	Herbivore	457	143	314
Artiodactyla	*Rusa unicolor* (Sambar)	Herbivore	272	123	149
Artiodactyla	*Moschus* spp.	Herbivore	246	125	121
Artiodactyla	*Naemorhedus caudatus* (Chinese goral)	Herbivore	115	27	88
Primates	*Macaca mulatta* (macaque)	Omnivore	576	234	342
Galliformes	*Ithaginis cruentus* (blood pheasant)	Herbivore	532	187	345
Galliformes	*Crossoptilon crossoptilon* (white-eared pheasant)	Herbivore	393	212	181
Total			8369	3173	5196

**Table 2:** Daily activity rhythm types of fifteen animals in the southwest mountains, China.

Species	RAI	Dawn (06:00–08:00)	Day (8:00–18:00)	Dusk (18:00–20:00)	Night (20:00–06:00)	Rhythm Types
Yellow-throated marten	0.36	10.45	80.60	7.46	1.49	Daytime
Macaque	3.06	7.99	80.38	6.94	4.69	Daytime
White-eared pheasant	2.02	13.74	70.48	12.72	3.05	Daytime
Blood pheasant	2.48	9.59	84.21	5.26	0.94	Daytime
Tufted deer	13.75	10.50	52.05	13.91	23.53	Mostly daytime
*Moschus* spp.	1.37	13.01	39.02	13.41	34.55	Mostly daytime
Wild boar	1.78	7.07	63.60	11.56	17.77	Mostly daytime
Leopard cat	0.50	5.48	5.48	13.70	75.34	Mostly nighttime
Asiatic black bear	0.24	17.39	4.35	8.70	69.57	Mostly nighttime
Red fox	1.05	8.37	23.72	13.02	54.88	Cathemeral
Sambar	1.58	5.51	22.43	14.71	57.35	Cathemeral
Chinese serow	1.40	8.97	25.16	12.25	53.61	Cathemeral
Chinese goral	1.11	12.17	34.78	2.61	50.43	Cathemeral
Leopard	0.27	0.00	34.62	11.54	53.85	Cathemeral
Eurasian lynx	0.66	15.15	21.21	9.09	54.55	Cathemeral

**Table 3:** Coefficients of daily activity overlapping (Δ1 and Δ4) and *p*-level (*p*) and their confidence intervals (CI) for predatory species.

Species 1	Species 2	Warm Season			Cold Season		
		Δ	CI	*p*	Δ	CI	*p*
Yellow-throated marten	White-eared pheasant	0.83	0.68–0.92	0.14	0.93	0.74–0.94	0.72
Yellow-throated marten	Blood pheasant	0.87	0.70–0.93	0.26	0.88	0.71–0.93	0.24
Yellow-throated marten	*Moschus* spp.	0.64	0.50–0.76	0	0.57	0.46–0.71	0
Yellow-throated marten	Tufted deer	0.76	0.63–0.85	0.01	0.71	0.60–0.80	0
Red fox	White-eared pheasant	0.6	0.50–0.72	0	0.42	0.37–0.53	0
Red fox	Blood pheasant	0.54	0.45–0.67	0	0.35	0.31–0.47	0
Red fox	*Moschus* spp.	0.68	0.59–0.81	0.14	0.76	0.67–0.85	0
Red fox	Tufted deer	0.78	0.68–0.86	0.10	0.6	0.55–0.69	0
Leopard cat	White-eared pheasant	0.35	0.24–0.51	0	0.27	0.22–0.41	0
Leopard cat	Blood pheasant	0.22	0.16–0.41	0	0.2	0.16–0.35	0

**Table 4:** Coefficients of daily activity overlapping (Δ1 and Δ4), with confidence intervals (CI) and *p*-levels (*p*) for competitor species.

Species 1	Species 2	Warm Season			Cold Season		
		Δ	CI	*p*	Δ	CI	*p*
Yellow-throated marten	White-eared pheasant	0.83	0.68–0.92	0.14	0.93	0.74–0.94	0.72
Yellow-throated marten	Blood pheasant	0.87	0.70–0.93	0.26	0.88	0.71–0.93	0.24
Yellow-throated marten	*Moschus* spp.	0.64	0.50–0.76	0	0.57	0.46–0.71	0
Yellow-throated marten	Tufted deer	0.76	0.63–0.85	0.01	0.71	0.60–0.80	0
Red fox	White-eared pheasant	0.6	0.50–0.72	0	0.42	0.37–0.53	0
Red fox	Blood pheasant	0.54	0.45–0.67	0	0.35	0.31–0.47	0
Red fox	*Moschus* spp.	0.68	0.59–0.81	0.14	0.76	0.67–0.85	0
Red fox	Tufted deer	0.78	0.68–0.86	0.10	0.6	0.55–0.69	0
Leopard cat	White-eared pheasant	0.35	0.24–0.51	0	0.27	0.22–0.41	0
Leopard cat	Blood pheasant	0.22	0.16–0.41	0	0.2	0.16–0.35	0

## Data Availability

The camera-trapping data are owned by the China West Normal University and are authorized for use in this study.

## Supplementary Materials

The following supporting information can be downloaded at: https://www.mdpi.com/article/10.3390/ani14192842/s1. Table S1: Coefficients of daily activity overlapping (Δ1 and Δ4), confidence intervals (CI) and p-level (p) for predatory species; Table S2: Coefficients of daily activity overlapping (Δ) and their confidence in-tervals (CI) of fifteen animals in southwest mountain, China; and Table S3: Coefficients of daily activity overlapping (Δ1 and Δ4), confidence intervals (CI) and p-level (p) for competitor species.
