# Supplementary material for: Daily Activity Rhythms of Animals in the Southwest Mountains, China: Influences of Interspecific Relationships and Seasons
Source: Animals (Basel). 2024 Oct 2;14(19):2842. doi: 10.3390/ani14192842 (PMC11476335; doi:10.3390/ani14192842)
Supplement: Supplementary file 1 [file animals-14-02842-s001.zip › animals-3224977-supplementary.pdf]

Table S1 Coefficients of daily activity overlapping (Δ1 and Δ4), confidence intervals (CI) and p-level (p) for predatory species

Species1	Species2	Warm Season			Cold Season		
		Δ	CI	p	Δ	CI	p
Wild boar	White eared pheasant	0.83	0.75-0.89	0.07	0.80	0.73-0.87	0
Wild boar	Blood pheasant	0.78	0.72-0.84	0	0.76	0.71-0.84	0
Asian black bear	White eared pheasant	0.35	0.22-0.55	0			
Asian black bear	Blood pheasant	0.26	0.16-0.45	0			
Eurasian lynx	White eared pheasant	0.47	0.33-0.63	0	0.43	0.26-0.65	0
Eurasian lynx	Blood pheasant	0.38	0.27-0.54	0	0.39	0.22-0.61	0
Eurasian lynx	Moschidae	0.60	0.46-0.75	0.15	0.78	0.54-0.86	0.24
Eurasian lynx	Tufted deer	0.61	0.47-0.75	0.04	0.63	0.44-0.80	0.02
Leopard	Tufted deer	0.51	0.26-0.66	0.09	0.59	0.46-0.68	0.01
Leopard	Moschidae	0.45	0.25-0.63	0.02	0.66	0.46-0.77	0.06
Leopard	Chinese serow	0.52	0.31-0.67	0.17	0.65	0.43-0.76	0.07
Leopard	Chinese goral	0.39	0.22-0.60	0.07	0.62	0.36-0.76	0.04
Leopard	Sambar	0.48	0.29-0.63	0.13	0.71	0.47-0.81	0.35
Leopard	Wild boar	0.48	0.24-0.65	0.04	0.55	0.42-0.68	0.01
Leopard	Red fox	0.49	0.26-0.64	0.09	0.61	0.38-0.74	0.14
Leopard	Yellow-throated marten	0.42	0.17-0.60	0.03	0.46	0.28-0.62	0.01
Leopard	Leopard cat	0.5	0.28-0.66	0.18	0.55	0.32-0.69	0.34
Leopard	White eared pheasant	0.45	0.19-0.60	0.04	0.38	0.28-0.52	0
Leopard	Blood pheasant	0.35	0.13-0.57	0.02	0.39	0.28-0.52	0

Table S2 Coefficients of daily activity overlapping (Δ) and their confidence intervals (CI) of fifteen animals in southwest mountain, China.

Species	Compared species													
Tufted deer	Tufted deer	Wild boar	Chinese serow	Sambar	Moschidae	Chinese goral	Red fox	Leopard cat	Yellow throated marten	Eurasian lynx	Leopard	Asiatic black bear	Macaque	Blood pheasant
Wild boar	0.85(0.83-0.89)	-												
Chinese serow	0.7(0.67-0.75）	0.62(0.58-0.69)	-											
Sambar	0.65(0.61-0.71）	0.57(0.52-0.65)	0.87(0.83-0.94)	-										
Moschidae	0.86(0.80-0.90)	0.73(0.68-0.80)	0.80(0.74-0.87)	0.75(0.68-0.82)	-									
Chinese goral	0.67(0.61-0.76)	0.62(0.55-0.73)	0.78(0.71-0.86)	0.68(0.63-0.81)	0.72(0.66-0.82)	-								
Red fox	0.68(0.64-0.76)	0.60(0.56-0.70)	0.94(0.86-0.95)	0.86(0.80-0.92)	0.78(0.72-0.87)	0.94(0.86-0.95)	-							
Leopard cat	0.47(0.44-0.59）	0.40(0.37-0.52)	0.76(0.70-0.85)	0.80(0.72-0.88)	0.60(0.54-0.72)	0.50(0.47-0.57)	0.78(0.69-0.85）	-						
Yellow throated marten	0.74(0.67-0.82)	0.83(0.74-0.89)	0.45(0.41-0.57)	0.41(0.36-0.53)	0.61(0.53-0.71)	0.41(0.39-0.49)	0.44(0.39-0.57)	0.23(0.20-0.40)	-					
Eurasian lynx	0.69(0.55-0.79)	0.60(0.47-0.73)	0.85(0.69-0.91)	0.81(0.66-0.90)	0.80(0.63-0.86)	0.78(0.71-0.86)	0.81(0.67-0.90)	0.72(0.58-0.85)	0.45(0.33-0.61)	-				
Leopard	0.68(0.53-0.78)	0.63(0.48-0.77)	0.74(0.60-0.85)	0.71(0.58-0.83)	0.68(0.54-0.79)	0.45(0.43-0.53)	0.71(0.56-0.82)	0.61(0.48-0.76)	0.49(0.34-0.66)	0.65(0.46-0.81)	-			
Asiatic black bear	0.50(0.40-0.68)	0.42(0.32-0.58)	0.75(0.58-0.85)	0.74(0.57-0.84)	0.62(0.49-0.78)	0.80(0.74-0.87)	0.75(0.58-0.86)	0.80(0.59-0.87)	0.27(0.18-0.47)	0.79(0.54-0.86)	0.49(0.31-0.70)	-		
Macaque	0.73(0.71-0.77)	0.83(0.78-0.87)	0.45(0.43-0.53)	0.40(0.37-0.48)	0.61(0.56-0.67)	0.75(0.58-0.85)	0.43(0.41-0.54)	0.24(0.22-0.36)	0.90(0.79-0.93)	0.45(0.34-0.60)	0.48(0.34-0.63)	0.28(0.20-0.46)	-	
Blood pheasant	0.69(0.67-0.73)	0.78(0.73-0.83)	0.41(0.39-0.49)	0.36(0.33-0.44)	0.57(0.53-0.65)	0.74(0.60-0.85)	0.40(0.37-0.50)	0.20(0.18-0.32）	0.91(0.78-0.94)	0.43(0.29-0.58)	0.48(0.34-0.63)	0.25(0.17-0.42)	0.89(0.85-0.94)	-
White eared pheasant	0.79(0.76-0.83)	0.82(0.77-0.87)	0.50(0.47-0.57)	0.45(0.41-0.53)	0.69(0.63-0.75)	0.85(0.69-0.91)	0.49(0.45-0.58)	0.29(0.26-0.41）	0.88(0.77-0.93)	0.51(0.38-0.64)	0.50(0.35-0.63)	0.33(0.23-0.51)	0.84(0.79-0.90)	0.85(0.81-0.91)

Table S3 Coefficients of daily activity overlapping (Δ1 and Δ4), confidence intervals (CI) and p-level (p) for competitor species

Species1	Species2	Warm Season			Cold Season		
		Δ	CI	p	Δ	CI	p
Wild boar	Macaques	0.85	0.77-0.89	0	0.82	0.75-0.87	0
Wild boar	Tufted deer	0.85	0.80-0.90	0	0.84	0.80-0.86	0
Wild boar	Chinese goral	0.6	0.51-0.71	0	0.62	0.46-0.87	0
Wild boar	Chinese serow	0.58	0.54-0.68	0	0.67	0.60-0.78	0
Wild boar	Moschidae	0.66	0.59-0.76	0	0.76	0.68-0.85	0
Wild boar	Sambar	0.62	0.55-0.72	0	0.52	0.46-0.63	0
Wild boar	Leopard cat	0.44	0.33-0.61	0	0.39	0.33-0.52	0
Wild boar	Yellow-throated marten	0.85	0.68-0.91	0.50	0.79	0.68-0.88	0.14
Wild boar	Red fox	0.71	0.60-0.83	0	0.54	0.49-0.66	0
White eared pheasant	Tufted deer	0.8	0.75-0.84	0	0.76	0.71-0.81	0
White eared pheasant	Sambar	0.43	0.37-0.53	0	0.46	0.40-0.57	0
White eared pheasant	Chinese serow	0.49	0.44-0.57	0	0.54	0.48-0.64	0
White eared pheasant	Chinese goral	0.5	0.43-0.62	0	0.48	0.34-0.67	0
White eared pheasant	Moschidae	0.69	0.60-0.77	0	0.64	0.56-0.72	0
Blood pheasant	Tufted deer	0.66	0.64-0.72	0	0.74	0.70-0.78	0
Blood pheasant	Chinese serow	0.38	0.35-0.47	0	0.48	0.43-0.59	0
Blood pheasant	Chinese goral	0.49	0.40-0.59	0	0.42	0.29-0.62	0
Blood pheasant	Sambar	0.32	0.28-0.42	0	0.42	0.36-0.53	0
Blood pheasant	Moschidae	0.53	0.49-0.64	0	0.59	0.51-0.68	0
Eurasian lynx	Leopard	0.28	0.12-0.53	0.08	0.62	0.31-0.78	0.11
